# Voriconazole Pharmacokinetics in Critically Ill Patients and Extracorporeal Membrane Oxygenation Support: A Retrospective Comparative Case-Control Study

**DOI:** 10.3390/antibiotics12071100

**Published:** 2023-06-25

**Authors:** Mar Ronda, Josep Manuel Llop-Talaveron, MariPaz Fuset, Elisabet Leiva, Evelyn Shaw, Victor Daniel Gumucio-Sanguino, Yolanda Diez, Helena Colom, Raul Rigo-Bonnin, Mireia Puig-Asensio, Jordi Carratalà, Ariadna Padullés

**Affiliations:** 1 Infectious Disease Department, Hospital Universitari de Bellvitge–IDIBELL, Hospitalet de Llobregat, 08907 Barcelona, Spain; maronse1@gmail.com (M.R.); evelyn.shaw@bellvitgehospital.cat (E.S.); mpuiga@bellvitgehospital.cat (M.P.-A.); jcarratala@bellvitgehospital.cat (J.C.); 2 Pharmacy Department, Hospital Universitari de Bellvitge–IDIBELL, Hospitalet de Llobregat, 08907 Barcelona, Spain; jmllopt@gmail.com (J.M.L.-T.); eleiva@bellvitgehospital.cat (E.L.); 3 Farmacoteràpia, Farmacogenètica i Tecnologia Farmacèutica, Hospital Universitari de Bellvitge–IDIBELL, Hospitalet de Llobregat, 08907 Barcelona, Spain; 4 Critical Care Department, Hospital Universitari de Bellvitge–IDIBELL, Hospitalet de Llobregat, 08907 Barcelona, Spain; mfuset@bellvitgehospital.cat (M.F.); vgumucio@bellvitgehospital.cat (V.D.G.-S.); 5 Centro de Investigación Biomédica en Red de Enfermedades Infecciosas (CIBERINFEC), Instituto de Salud Carlos III, 28019 Madrid, Spain; 6 Epidemiologia de les Infeccions Bacterianes, Patologia Infecciosa i Transplantament, Hospital Universitari de Bellvitge–IDIBELL, Hospitalet de Llobregat, 08907 Barcelona, Spain; 7 Hospital Universitari de Bellvitge–IDIBELL, Hospitalet de Llobregat, 08907 Barcelona, Spain; ydiez@bellvitgehospital.cat; 8 Biopharmaceutics and Pharmacokinetics Unit, Department of Pharmacy and Pharmaceutical Technology and Physical-Chemistry, School of Pharmacy, University of Barcelona, 08028 Barcelona, Spain; helena.colom@ub.edu; 9 Clinical Laboratory, Hospital Universitari de Bellvitge–IDIBELL, Hospitalet de Llobregat, 08907 Barcelona, Spain; raulr@bellvitgehospital.cat; 10 Department of Clinical Sciences, Faculty of Medicine and Health Sciences, University of Barcelona, 08036 Barcelona, Spain

**Keywords:** voriconazole, extracorporeal membrane oxygenation, therapeutic drug monitoring

## Abstract

Voriconazole, an antifungal agent, displays high intra- and inter-individual variability. The predictive pharmacokinetic (PK) index requires a minimum plasma concentration (C_min_) in patient serum of between 1–5.5 mg/L. It is common to encounter fungal infections in patients undergoing extracorporeal membrane oxygenation (ECMO) support, and data regarding voriconazole PK changes during ECMO are scarce. Our study compared voriconazole PKs in patients with and without ECMO support in a retrospective cohort of critically-ill patients. Fifteen patients with 26 voriconazole C_min_ determinations in the non-ECMO group and nine patients with 27 voriconazole C_min_ determinations in the ECMO group were recruited. The ECMO group had lower C_min_ (0.38 ± 2.98 vs. 3.62 ± 3.88, *p* < 0.001) and higher infratherapeutic C_min_ values (16 vs. 1, *p* < 0.001) than the non-ECMO group. Multivariate analysis identified ECMO support (−0.668, CI_95_ −0.978–−0.358) and plasma albumin levels (−0.023, CI_95_ −0.046–−0.001) as risk factors for low C_min_ values. When comparing pre- and post-therapeutic drug optimisation samples from the ECMO group, the dose required to achieve therapeutic C_min_ was 6.44 mg/kg twice a day. Therapeutic drug optimisation is essential to improve target attainment.

## 1. Introduction

The emergence of the COVID-19 pandemic resulted in a higher incidence of severe acute respiratory distress syndrome (ARDS). As part of the therapeutic strategy for ARDS, extracorporeal membrane oxygenation (ECMO) support is employed when conventional measures fail [1]. It is well-known that ECMO and critically-ill patients are at higher risk of contracting nosocomial infections [2], with the most commonly reported invasive life-threatening fungal diseases being caused by *Aspergillus* spp. In addition, COVID-19 patients have a higher risk of contracting COVID-19-associated pulmonary aspergillosis (CAPA), an invasive infection caused by *Aspergillus* spp. [3,4].

In critically-ill patients with COVID-19-associated ARDS, distinguishing between Aspergillus colonisation and CAPA is challenging due to the non-specific nature of radiological imaging [3,5] and the lack of established galactomannan cut-off points for non-neutropenic patients [6]. Furthermore, a recent meta-analysis demonstrated a 25% higher mortality rate in CAPA patients compared with critically-ill COVID-19 patients without Aspergillus [7].

The first-line therapy in patients with a CAPA diagnosis consists of either voriconazole or isavuconazole [5]. Voriconazole is a second-generation triazole that exhibits a non-linear dose-dependent pharmacokinetic (PK) profile [8]. The standard dosing for intravenous administration is a loading dose of 6 mg/kg twice a day (BID), followed by a maintenance dose of 4 mg/kg BID [9]. When calculating voriconazole dosages, it is important to consider the patient’s body mass index (BMI). Previous studies have shown that in patients with a BMI of ≥ 30 kg/m^2^, voriconazole should be dosed based on the adjusted body weight (ABW), calculated as described elsewhere, using a conversion factor of 0.4 [10,11].

Voriconazole presents time-dependent fungicidal activity against *Aspergillus* spp., and its predictive PK index is the minimum plasma concentration (C_min_) in patient serum, with an optimal C_min_ range of between 1 mg/L and 5.5 mg/L [12]. Due to its non-linear PKs and high intra- and inter-individual variability [13], combined with its narrow therapeutic range, all clinical guidelines strongly recommend therapeutic drug monitoring (TDM) [14,15].

This antifungal agent has also been seen to undergo multiple interactions, which have been widely defined. On one hand, some biological variables presumed to modify voriconazole plasma levels are inflammation determined by C-reactive protein [16], bilirubin levels [17,18], the CYP2C19 genotype, which can induce ultra-rapid voriconazole metabolism [19], or other numerous drug-drug interactions such as protein pump inhibitors (PPI) and corticosteroids [12,20].

On the contrary, data regarding PK changes in patients undergoing ECMO are limited, but some studies have shown that ECMO significantly alters the PK profile of voriconazole, which can lead to therapeutic failure [21,22]. Voriconazole is a lipophilic drug with high protein binding (60%), which induces drug sequestration in the components of the circuit, thereby increasing its distribution volume. Another factor that alters voriconazole PKs is the duration of membrane use, due to the eventual saturation of its adsorption sites. The number of days until the membrane collapses has yet to be defined, however, it can be determined by the membrane’s ability to oxygenate and changes in drug plasma concentration [21,23].

This study aims to compare voriconazole PKs in patients receiving ECMO support versus those who do not and determine the optimal voriconazole dosage required to achieve and maintain the PK target in a timely manner.

## 2. Results

### 2.1. Patients and Characteristics

A total of 24 ICU patients treated with voriconazole were included in the study, 9 in the ECMO group and 15 in the non-ECMO group. Most patients were men (62.5%) with a median age of 58 ± 10 years and a BMI of 28.3 ± 5.3 kg/m^2^. None of the patients were undergoing renal replacement therapy, and 15 patients (62.5%) dsied at the end of the episode. No significant differences in baseline demographic parameters were observed between ECMO and non-ECMO patients; however, patients receiving ECMO support were treated with voriconazole for longer than those without ECMO support (38 ± 17 versus 11 ± 10, *p* = 0.003) and underwent more C_min_ determinations during treatment (3 ± 2 versus 1 ± 0.5, *p* = 0.005). Most patients in the ECMO-support group were diagnosed with CAPA (78%) (see Table 1).

### 2.2. Extracorporeal Membrane Oxygenation

All patients in the ECMO group received venous–venous support for a median of 47 ± 24.25 days. The most used blood pump was a Maquet Rotaflow (4, 50%), and the most frequently used membrane oxygenator was a Quadrox PLS (Maquet) (3, 37.5%). During voriconazole treatment, ECMO membrane changes were performed on 10 occasions in six patients, while three patients did not undergo an ECMO circuit exchange. The C_min_ values after the saturation effect resulting from the collapse of all active membrane sites were only determined in one patient (Figure S1). Samples were obtained with a median blood pump speed of 3700 ± 1475 rpm and flux of 4.75 ± 2.00 mL/min (Table S1).

### 2.3. Voriconazole Sample Determinations

During the study period, 53 samples were collected, 27 from the ECMO group and 26 from the non-ECMO group. Most patients were treated with intravenous voriconazole (30, 57%). On average, 2.21 voriconazole C_min_ measurements were obtained per patient, with tighter C_min_ control observed in patients with ECMO support than those without (3.00 ± 2 versus 1.00 ± 0.5, respectively).

Statistically significant differences were observed between the non-ECMO and ECMO groups for the median standardised voriconazole dose on sampling days (3.53 versus 4.50 mg/kg BID, respectively, *p* = 0.007), being significantly higher in the ECMO group. In the non-ECMO versus the ECMO group, the median C_min_ (3.62 versus 0.38 mg/L, respectively, *p* < 0.001) and median C_min_/dose (0.98 versus 0.12 kg/L, respectively, *p* < 0.001) were significantly lower.

Significant differences in biochemical variables between the groups were only found for albumin (*p* = 0.02) and gamma-glutamyl transferase (GGT) (*p* = 0.001), both being higher in the ECMO group. None of the pharmacological interactions described in the literature (PPI, corticosteroids, and amiodarone) presented statistically significant differences between the groups (Table 1).

After screening all relevant biologically plausible covariates that could influence voriconazole exposure, significant differences were found in the univariate analysis for age, BMI, albumin, ECMO support, and type of support (oxygenator and flow speed) (Table S2). However, only ECMO support and albumin remained significant in the multivariate analysis after adjusting the model for time period (Table 2).

### 2.4. ECMO Subgroup Analysis

**Figure 1:**
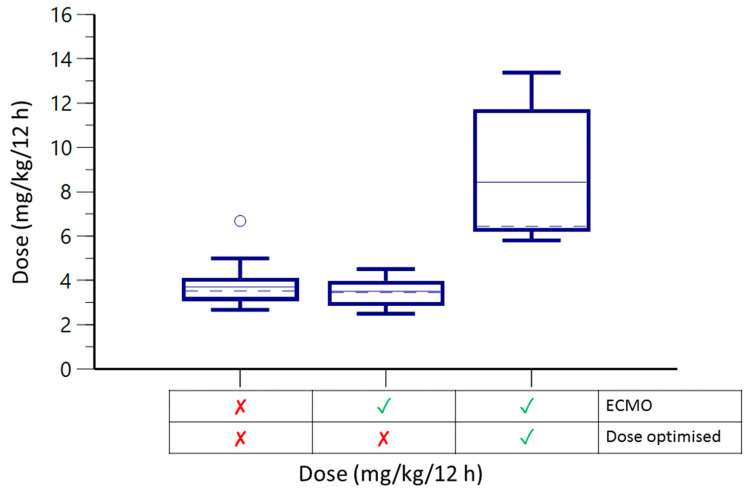
Voriconazole doses according to dose strategy and group. The checkmark (✓) signifies the presence of ECMO or dose optimization, whereas the red cross (✘) indicates its absence. The line enclosed within the box represent quartile 1, 2 and 3. The lower line denotes the minimum value, while the upper line represents the maximum value. Lastly, the singular point symbolizes an outlier.

In the secondary analysis, we observed that the non-optimised subgroup samples were infra-therapeutic in 88% of cases, with a median C_min_ of 0.30 ± 0.06 mg/L while receiving a median dose of 3.48 ± 1.13 mg/kg BID. After dose optimisation, 56% of voriconazole C_min_ values were within the therapeutic range (median C_min_ 3.28 ± 3.09 mg/L), with a median dose of 6.44 ± 5.51 mg/kg BID (Table 3). Hepatic enzymes remained stable after increasing the dose, except for alanine transferase (ALT) (34.5 versus 77 U/L, respectively, *p* = 0.011). Only one patient from each subgroup was a CAPA survivor. The voriconazole doses received according to the dose adjustment strategy are shown in Figure 1.

## 3. Discussion

Our study demonstrates that TDM of voriconazole is a crucial tool to ensure that drug plasma levels fall within the effective range in patients receiving ECMO support. We found that a maintenance dose of 6 mg/kg BID is needed to achieve the optimal PK target in these patients, which is higher than standard dosing. Additionally, our study identified ECMO support and plasma albumin concentrations as independent variables influencing voriconazole C_min_ values.

Many single-patient case reports have been published in recent years regarding the fluctuation of plasma voriconazole concentrations in patients receiving ECMO support [21,22,24,25,26,27,28]; however, only two major retrospective studies have been published [29,30]. All these studies reported very heterogeneous results, probably due to various factors such as ECMO circuitry, drug-drug interactions, and other physiological factors that should be considered to influence voriconazole PKs.

### 3.1. Variables Affecting Voriconazole C_min_

As previously mentioned, ECMO can lead to changes in voriconazole PKs by increasing the distribution volume, decreasing antimicrobial elimination, and sequestering the drug in its circuitry; however, the magnitude of these modifications has not been quantified [23]. A recent ex vivo study demonstrated significant drug loss through the ECMO circuitry due to the high lipophilicity of the molecule [31]. In fact, our experience showed higher variations in voriconazole C_min_ after changing the ECMO membrane (Figure S1), as suggested by Winiszewski et al., [24] and Mathieu et al., [25]. In contrast, Van Daele et al., did not demonstrate any impact of ECMO support on voriconazole exposure [29], nor did the case report of Lin et al., [27]. In the former, this may be because most C_min_ values in the non-ECMO branch were obtained from one centre (128 out of 147) and the control group was derived from pre- and post-ECMO periods, as well as the bias of post-ECMO determinations based on the longer voriconazole treatment and increasing doses guided by TDM [29], although ECMO circuitry features may play a significant role in voriconazole PKs. Also, the recent retrospective cohort comparing voriconazole PKs in patients with and without ECMO support published by Ye et al., showed that the ECMO group presented infratherapeutic C_min_ values in 51% of occasions, a percentage similar to ours, with ECMO support being the main reason for these infratherapeutic values. However, they also observed that the ECMO flow rate has a negative correlation with plasma voriconazole C_min_ levels, which we failed to demonstrate [30].

On the other hand, the impact of hypoalbuminemia on voriconazole PKs has been extensively described in the literature [13,32,33]. The non-linear PK profile of voriconazole prevents the elimination of the drug when high-unbound drug levels are present in plasma due to lower plasma protein binding [17], which is consistent with the findings of our study. A relatively recent study by Chantharit et al., suggested that patients with lower albumin levels have a lower voriconazole clearance, using a cut-off value of 30 g/L for albumin [32].

In our study, previously described biological variables modifying voriconazole PKs did not present significant effects on voriconazole C_min_. One possible explanation for the lack of significance of PPI treatment in our study is that almost all patients were already undergoing PPI treatment (98%), and the PPI dosage was not taken into account as some studies suggest that cut-off dosages of 20 mg can induce significant differences in voriconazole C_min_ [13]. Corticosteroids are known to interact with voriconazole [34,35], however, our study did not identify them as significant modifiers, possibly due to the small number of samples obtained from patients on corticosteroid treatment (37%) or the inclusion of all types of glucocorticoids (i.e., dexamethasone, methylprednisolone, and prednisone) without dose distinction, factors which have been determined as key elements for determining the PK influence [36].

### 3.2. Antifungal Recommendations in Patients with ECMO Support

Voriconazole is the gold standard therapy for treating CAPA [37,38]. Patients with infratherapeutic voriconazole C_min_ are more likely to have unsuccessful outcomes without differences in mortality, while patients with supratherapeutic C_min_ are prone to toxicity [18,39]. In our study we demonstrated that the subgroup analysis of patients under ECMO support, we demonstrated that higher than standard doses are required to achieve the objective C_min_. In fact, new TDM guidelines recommend voriconazole C_min_ values of 2–6 mg/L for severe infections with close control of hepatic and neurological toxicity [15], supporting our findings on increasing voriconazole doses.

Voriconazole toxicity presents as hepatotoxicity, defined as an increase in liver transaminase levels, and neurotoxicity, including encephalopathy (characterised by confusion and agitation), extrapyramidal signs, myoclonus, and auditory and visual hallucinations [40]. Monitoring the neurological toxicity of voriconazole in critically-ill patients can be challenging as they are often sedated, therefore, focusing on monitoring liver function is recommended in these patients. In our study, we found that after dose optimisation of up to 6 mg/kg BID, only a mild increase in ALT was observed, suggesting the absence of hepatic toxicity and the safety of increasing voriconazole doses.

Another first-line therapy for CAPA is isavuconazole, the newest agent of its class [38,39]. In this case, the effect that ECMO and other supportive techniques induces on their plasma concentrations is still not clear, so TDM is also recommended in such cases [41,42]. However, plasma concentrations and PK determination of isavuconazole are less available [43], and data about ECMO support and TDM in those cases are scarce [44].

Second-line treatment experiences have also been described as viable alternatives. These options encompass liposomal amphotericin B or the combination of azoles and echinocandins [5,38,39]. The utilisation of liposomal amphotericin B in ECMO patients has been documented in a limited number of cases, yielding contradictory findings [44]. Although the efficacy of both azoles and liposomal amphotericin B is relatively comparable, the adverse event profile of liposomal amphotericin B relegates it to a second-line therapy [5,45]. All other antifungals and their combinations are regarded as salvage therapy in instances of refractory invasive aspergillosis or intolerance to the other approved treatments [45].

### 3.3. Strengths and Limitations

To the best of our knowledge, this is the second-largest retrospective real-life study of voriconazole PKs in patients receiving ECMO support. Furthermore, the generalised estimating equations (GEE) applied to study the association between C_min_/daily dose/kg BID and previously statistically significant variables obtained in the univariate analysis provide the highest consistent estimate of outcomes, which is more robust to population normality violations or possible covariance. However, several limitations should be acknowledged. First, the retrospective design and lack of randomisation could introduce confounding factors and limit the ability to establish causality. In addition, the fact that CYP2C19 genotyping was not performed in all patients means that the impact of this genetic factor on voriconazole metabolism could not be fully evaluated [19]. Also, the sample was too small to detect differences in clinical outcomes such as all-cause mortality and CAPA-related mortality, which was not an aim of this study. Lastly, the lack of matching between ECMO and non-ECMO groups could introduce selection bias. All those limitations may restrict the generalisability of the findings.

## 4. Materials and Methods

### 4.1. Design and Setting

A retrospective, observational, single-centre study was conducted at Bellvitge University Hospital, a tertiary-level hospital, between 1 November 2012 and 1 February 2022.

All adult patients admitted to the intensive care unit (ICU) and treated with voriconazole, who had at least one C_min_ determination as part of routine clinical practice, were eligible for inclusion in the study, regardless of the type of treatment indication (empirical or targeted) or the route of administration (oral, nasogastric tube, or intravenous). The patients were divided into two groups depending on whether they were receiving ECMO support (ECMO group) or not (non-ECMO) while undergoing voriconazole treatment.

As per protocol, the first plasma concentration was determined five to seven days after treatment initiation, when steady-state conditions were achieved. Blood samples were taken 15 to 60 min prior to the next dose (C_min_). Plasma voriconazole concentrations were measured using a validated high-pressure liquid chromatography (HPLC) method as previously published [46]. The voriconazole C_min_ was considered adequate when ranging between 1 and 5.5 mg/L, as described in previous studies [12]. In cases where C_min_ values were ≤0.3 mg/L, they were assumed to be equal to 0.3 mg/L for the purposes of calculations. When suboptimal C_min_ values were detected, drug optimisation was implemented according to local clinical practice guidelines applied in daily standard practice.

A secondary analysis was conducted on the ECMO group, differentiating voriconazole C_min_ determinations before and after TDM and dose optimisation. These patients were divided into two subgroups: a non-optimised group comprising patients who received voriconazole doses of <5 mg/kg BID, and an optimised group that received voriconazole doses of ≥5 mg/kg BID.

### 4.2. Data Sources and Collection

All data were collected from electronic medical records and included patient demographics (age, gender, weight, height, BMI), glomerular filtration rate (measured using the CKD-EPI formula) and renal replacement therapy, biochemical parameters including albumin, C-reactive protein (CRP), direct bilirubin, GGT, alkaline phosphatase (APT), aspartate transaminase (AST), ALT, type and duration of ECMO support, indications and settings for ECMO (type of oxygenator, blood pump and its speed and flux), voriconazole dosage and C_min_, concomitant medications, drug-drug interactions (such as PPI, corticosteroids, and other literature-described CYP2C19 inducers), CAPA diagnosis, and all-cause mortality during the episode for which the patient was included.

Voriconazole dosage was standardised according to actual body weight for patients with a BMI <30 kg/m^2^ and using an ABW calculated by applying a 0.4 factor for patients with a BMI ≥30 kg/m^2^. The concentrations determined were considered actual C_min_ if collected between five and thirty minutes before dose administration. Post-dose plasma voriconazole concentrations were excluded from the analysis.

### 4.3. Statistical Methods

Continuous variables were described using the median and interquartile range (IQR), and categorical variables were described as numbers and percentages. Voriconazole C_min_ values were normalised per dose and body weight (C_min_/daily dose/kg BID). Normal distribution was assessed using the Kolmogorov-Smirnov test. For the univariate approach, we studied the association between categorical variables using the chi-square test with Fischer’s exact test. The association between categorical and continuous variables was determined using the Student’s *t*-test and one-way analysis of variance (F distribution). The association between continuous variables was stated by using simple linear regression models. A *p*-value of <0.05 based on a two-sided test was considered statistically significant. Variables included in the univariate analysis were those previously reported in the literature to influence voriconazole PKs. GEEs were applied to study the association between C_min_/daily dose/kg BID and previously statistically significant variables obtained in the univariate analysis. The factors were assumed to be categorical and covariates were assumed to be scaled. Statistical analyses were performed using SPSS statistics version 29.0 (SPSS Inc., an IBM Company, Chicago, IL, USA).

## 5. Conclusions

This study demonstrates that the voriconazole dose required to attain the objective PKs in patients receiving ECMO support is higher than that recommended elsewhere. Due to the significant inter- and intra-individual variability of the drug, we observed that a maintenance dose of 6 mg/kg BID voriconazole combined with TDM is required to achieve therapeutically effective and safe plasma levels, with a lower incidence of adverse events. Additionally, our study confirms the importance of monitoring albumin levels and adjusting the voriconazole C_min_ obtained to prevent toxicity. Randomised clinical trials are required to determine the clinical relevance in terms of mortality.

## Figures and Tables

**Table 1:** Voriconazole trough samples in the non-ECMO and ECMO groups.

Therapeutic Drug Monitoring	Total (N = 53)	Non-ECMO Group (N = 26)	ECMO Group (N = 27)	*p*-Value
**Demographics**				
Age (years)	58 (10)	65 (18)	58 (2)	0.115
Gender (women)	9 (37.5)	5 (33.3)	4 (44.4)	0.678
BMI (kg/m^2^)	28.3 (5.3)	25.3 (5.1)	30 (1.8)	0.050
Actual weight (kg)	72.5 (11.5)	70 (15)	75 (12)	**0.034**
Adjusted weight (kg)	64.6 (11.2)	62.6 (14.4)	66.6 (9.4)	0.272
Days of treatment	16.5 (28)	11 (12)	38 (24)	**0.003**
Number of C_min_ determinations per patient	1 (3)	1 (1)	4 (2)	**0.005**
CAPA diagnosis	8 (35)	1 (7)	7 (78)	NR
Mortality (yes) *	15 (62.5)	8 (53.3)	7 (77.8)	0.389
**Drug parameters**				
C_min_ (mg/L)	2.12 (4.08)	3.62 (3.88)	0.38 (2.98)	**<0.001**
Standardised doses (mg/kg/12 h)	4.00 (1.63)	3.53 (1.04)	4.50 (3.10)	**0.007**
C_min_/daily dose/kg ((mg/L)/(kg/day))	0.41 (0.93)	0.98 (1.12)	0.12 (0.33)	**<0.001**
Number of infra-therapeutic samples (C_min_ < 1)	17 (32.1)	1 (3.8)	16 (59.3)	**<0.001**
Number of therapeutic samples (C_min_ 1–5)	26 (49.1)	16 (61.5)	10 (37.0)	
Number of supra-therapeutic samples (C_min_ > 5)	10 (18.9)	9 (34.6)	1 (3.7)	
Proton pump inhibitors	52 (98.1)	25 (96.2)	27 (100)	0.304
Corticosteroids	24 (45.3)	15 (57.7)	9 (33.3)	0.075
Amiodarone ^1^	5 (9.4)	5 (19.2)	0 (0)	**0.017**
**Analytical variables**				
Renal clearance (CDK-EPI, mL/min)	110.20 (49.32)	99.98 (48.19)	147.60 (27.50)	**<0.001**
C-reactive protein (mg/L)	98.20 (117.90)	78.90 (103.95)	116.70 (85.00)	0.374
Alanine transferase (U/L)	42.00 (58.20)	39.30 (54.75)	51.00 (61.00)	0.910
Aspartate transferase (U/L)	39.30 (40.20)	34.50 (28.95)	50.50 (58.25)	0.051
Gamma-glutamyl transferase (U/L)	334.00 (730.00)	97.8 (303.75)	614.00 (1322.00)	**0.001**
Bilirubin (μmol/L)	8.00 (8.00)	8.00 (7.25)	8.00 (8.00)	0.971
Alkaline phosphatase (U/L)	154.00 (190.94)	122.98 (86.20)	226.00 (210.57)	0.073
Albumin (g/L)	29.00 (9.00)	27.50 (6.50)	30.00 (11.00)	**0.020**

^1^ All samples were obtained from only three patients: three determinations from one patient and one from two patients. * One patient from the non-ECMO group was lost to follow-up; CAPA: SARS-CoV-2-associated Aspergillosis; NR: not relevant. Statistically significant values are marked in bold.

**Table 2:** Effect of variables on voriconazole C_min_ using the generalised estimating equations (GEE) model.

**Outcomes**	**Beta**	**95% CI**	***p*-Value**
Albumin (g/L)	−0.023	−0.046–−0.001	**0.044**
ECMO support	−0.668	−0.978–−0.358	**<0.001**
Corticosteroids	−0.026	−0.046–−0.001	0.908

Statistically significant values are marked in bold.

**Table 3:** Differences between optimised and non-optimised voriconazole samples in the ECMO group.

	Non-Optimised ECMO (N = 16)	Optimised ECMO (N = 11)	*p*-Value
Standardised doses (mg/kg/12 h)	3.48 (1.13)	6.44 (5.51)	**<0.001**
C_min_ (mg/L)	0.30 (0.06)	3.28 (3.09)	**0.002**
C_min_/daily dose/kg ((mg/L)/(kg/day))	0.10 (0.04)	0.35 (0.50)	**0.200**
Number of infra-therapeutic samples (C_min_ < 1)	14 (87.5)	2 (18.2)	**<0.001**
Number of therapeutic samples (C_min_ 1–5)	1 (6.3)	9 (81.8)	
Number of supra-therapeutic samples (C_min_ > 5)	1 (6.3)	0	
Alanine transferase (U/L)	34.50 (32.25)	77.00 (48.00)	**0.011**
Aspartate transferase (U/L)	38.99 (48.00)	64.00 (79.00)	0.254
Gamma-glutamyl transferase (U/L)	511.00 (591.25)	1031.00 (1790.00)	0.353
Bilirubin (μmol/L)	8.00 (8.00)	10.00 (9.00)	0.831
Alkaline phosphatase (U/L)	216.50 (227.60)	255.00 (221.00)	0.327

Statistically significant values are marked in bold.

## Data Availability

The datasets used and analysed during the current study are available from the corresponding author upon reasonable request.

## Supplementary Materials

The following supporting information can be downloaded at: https://www.mdpi.com/article/10.3390/antibiotics12071100/s1; Table S1: ECMO device variables; Table S2: Univariate analysis; Figure S1: Evolution of voriconazole dosages and C_min_ plasma concentrations in a patient with wild-type CYP2C19 during ECMO support in the ICU. The red dots represent voriconazole doses expressed as mg/kg of adjusted weight administered every 12 h, while blue dots indicate the corresponding plasma concentrations of voriconazole. Membrane exchanges are indicated with arrows.
