# Supplementary material for: Voriconazole Pharmacokinetics in Critically Ill Patients and Extracorporeal Membrane Oxygenation Support: A Retrospective Comparative Case-Control Study
Source: Antibiotics (Basel). 2023 Jun 25;12(7):1100. doi: 10.3390/antibiotics12071100 (PMC10376825; doi:10.3390/antibiotics12071100)
Supplement: Supplementary file 1 [file antibiotics-12-01100-s001.zip › antibiotics-2442078-supplementary.pdf]

antibiotics

MDPI

Supplementary materials

Table S1. ECMO device variables.

**ECMO group** (N = 9 patients)		
Type of ECMO	Venous-venous	9 (100)
	Venous-arterial	0 (0)
Oxygenator membrane*	EOS ECMO (Sorin)	1 (12.50)
	Quadrox PLS (Maquet)	3 (37.50)
	Euroset	2 (25.00)
	HLS 5 (Maquet)	1 (12.50)
	HLS 7 (Maquet)	1 (12.50)
Blood pump*	Maquet Rotaflow	4 (50.00)
	Centrimag and Pedimag	2 (25.00)
	Cardiohelp	2 (25.00)
Days of ECMO support		47 (24.25)
Number of membrane exchanges		10 (37.00)
**ECMO group** (N = 27 samples)		
ECMO FiO_2 (%)		100 (10.00)
Blood pump speed (rpm)		3700 (1475.00)
Blood pump flux (mL/min)		4.80 (2.00)

*Data missing from one patient.

Table S2. Univariate analysis.

Variable	*b* Value	R2 Adjusted Value	*p*-value
C-reactive protein (mg/L)	0.000	−0.015	0.636
Albumin (g/L)	−0.047	0.187	<0.001
Age	0.019	0.067	0.034
BMI (kg/m2)	−0.06	0.079	0.024
ECMO support	-	-	0.001
Blood pump flux (mL/min)	−0.051	−0.017	0.456
Blood pump speed (rpm)	0.000	0.154	0.024

b Value is the regression coefficients for the corresponding independent variable, R2 Adjusted Value explains the proportion of variability in the dependent variable.

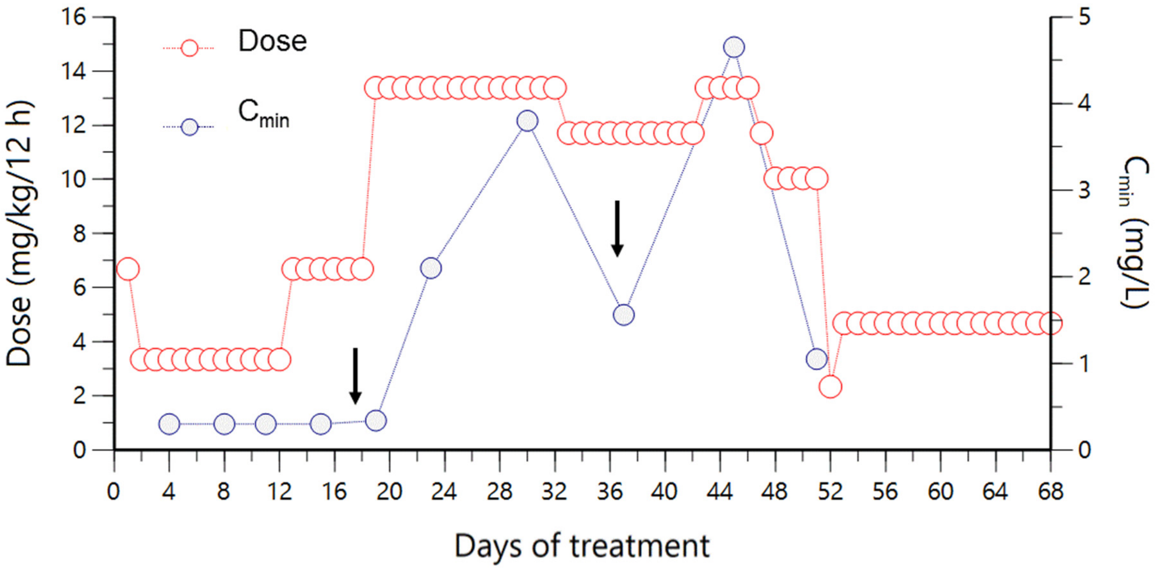

Figure S1. Evolution of voriconazole dosages and C_{min} plasma concentrations in a patient with wild-type CYP2C19 during ECMO support in the ICU. The red dots represent voriconazole doses expressed as mg/kg of adjusted weight administered every 12 hours, while blue dots indicate the corresponding plasma concentrations of voriconazole. Membrane exchanges are indicated with arrows.
